# Imprinting methylation in *SNRPN* and *MEST1* in adult blood predicts cognitive ability

**DOI:** 10.1371/journal.pone.0211799

**Published:** 2019-02-01

**Authors:** Marlene Lorgen-Ritchie, Alison D. Murray, Anne C. Ferguson-Smith, Marcus Richards, Graham W. Horgan, Louise H. Phillips, Gwen Hoad, Ishbel Gall, Kristina Harrison, Geraldine McNeill, Mitsuteru Ito, Paul Haggarty

**Affiliations:** 1 Rowett Institute of Nutrition and Health, University of Aberdeen, Aberdeen, United Kingdom; 2 Aberdeen Biomedical Imaging Centre, University of Aberdeen, Aberdeen, United Kingdom; 3 Department of Genetics, University of Cambridge, Cambridge, United Kingdom; 4 MRC Unit for Lifelong Health and Ageing, University College London, London, United Kingdom; 5 Biomathematics and Statistics Scotland, University of Aberdeen, Aberdeen, United Kingdom; 6 School of Psychology, University of Aberdeen, Aberdeen, United Kingdom; 7 Department of Pathology, N.H.S. Grampian, Aberdeen, United Kingdom; 8 Institute of Applied Health Sciences, University of Aberdeen, Aberdeen, United Kingdom; University of Missouri Columbia, UNITED STATES

## Abstract

Genomic imprinting is important for normal brain development and aberrant imprinting has been associated with impaired cognition. We studied the imprinting status in selected imprints (*H19*, *IGF2*, *SNRPN*, *PEG3*, *MEST1*, *NESPAS*, *KvDMR*, *IG-DMR* and *ZAC1*) by pyrosequencing in blood samples from longitudinal cohorts born in 1936 (n = 485) and 1921 (n = 223), and anterior hippocampus, posterior hippocampus, periventricular white matter, and thalamus from brains donated to the Aberdeen Brain Bank (n = 4). *MEST1* imprint methylation was related to childhood cognitive ability score (-0.416 95% CI -0.792,-0.041; p = 0.030), with the strongest effect evident in males (-0.929 95% CI -1.531,-0.326; p = 0.003). *SNRPN* imprint methylation was also related to childhood cognitive ability (+0.335 95%CI 0.008,0.663; p = 0.045). A significant association was also observed for *SNRPN* methylation and adult crystallised cognitive ability (+0.262 95%CI 0.007,0.517; p = 0.044). Further testing of significant findings in a second cohort from the same region, but born in 1921, resulted in similar effect sizes and greater significance when the cohorts were combined (*MEST1*; -0.371 95% CI -0.677,-0.065; p = 0.017; *SNRPN*; +0.361 95% CI 0.079,0.643; p = 0.012). For *SNRPN* and *MEST1* and four other imprints the methylation levels in blood and in the five brain regions were similar. Methylation of the paternally expressed, maternally methylated genes *SNRPN* and *MEST1* in adult blood was associated with cognitive ability in childhood. This is consistent with the known importance of the *SNRPN* containing 15q11-q13 and the *MEST1* containing 7q31-34 regions in cognitive function. These findings, and their sex specific nature in *MEST1*, point to new mechanisms through which complex phenotypes such as cognitive ability may be inherited. These mechanisms are potentially relevant to both the heritable and non-heritable components of cognitive ability. The process of epigenetic imprinting—within *SNRPN* and *MEST1* in particular—and the factors that influence it, are worthy of further study in relation to the determinants of cognitive ability.

## Introduction

Human cognitive ability is an important determinant of educational and occupational success, social mobility, health, and longevity [1] though it is not clear whether higher cognitive ability leads to better health and longevity through improved lifestyle choices and life opportunities or whether there is a common biological basis to a well-functioning brain and body. In addition to uncertainty over the direction of causality, the transgenerational nature of these links suggests that causal pathways may span more than one generation. Cognitive ability is influenced both by genetics and the environment [1]. Epigenetic states are generally erased between generations. However, examples of heritability from parent to offspring with possible relevance to transgenerational transmission of phenotypes have been reported in mouse and human [2,3].

One class of epigenetics—imprinting—is particularly relevant to transgenerational and early life effects since imprints are established in the germline, maintained during the preimplantation reprogramming phase, and then passed on through the somatic cell lineages, where they can influence genome function and gene expression [4]. The sensitivity of some imprints to environmental effects prior to birth [5–8] suggests the potential for epigenetic states and their related biological functions to also be influenced by the early environment in a way that may persist in later life. Imprints as a class are also generally stable over time, and for some imprints the original signal persists in a wide range of cell types many divisions and decades later [9,10]. These characteristics make imprints particularly amenable to study in longitudinal cohort designs where only blood samples may be available and it may not be possible to sample the tissues, such as the brain, which is the primary interest in the case of cognitive phenotypes. Imprints are particularly promising candidates in the study of cognition as they are known to be important for neurogenesis, brain function and behaviour [11–15], and they have even been proposed as possible mediators of reported generational increases in intelligence test scores [16].

This study investigates the link between epigenetic imprinting and cognitive ability at age 11 and in adulthood using data from a well-characterised cohort born in 1936 and recruited at 64 years of age. A second cohort born in the same geographical region in 1921 and recruited at 78 years of age was used to further assess significant findings in the 1936 cohort. We focused on nine selected imprints (*H19*, *IGF2*, *SNRPN*, *PEG3*, *MEST1*, *NESPAS*, *KvDMR*, *IG-DMR* and *ZAC1*). All except *IGF2* are germline imprints [17]. These genes, or the chromosomal regions containing them, have been implicated in cognitive development, or early life outcomes such as birth weight which have been related to cognitive outcomes, in humans and animal models [8,18–24]. We also compared the methylation level in the blood and selected regions of human brain sampled post-mortem for each imprint. The blood methylation levels were related to cognitive ability in childhood in the cohort born in 1936 and significant results were further evaluated using the cohort born in 1921.

## Materials and methods

Participants were members of the Aberdeen Birth Cohorts of 1936 (ABC36) and 1921 (ABC21) from whom DNA was collected (n = 485 and n = 223, respectively) [25]. Ethical approval for the study was obtained from the Multi-Centre Research Ethics Committee for Scotland (MREC/01/0/56) and Grampian Research Ethics Committee (LREC/01/0299). The research was conducted in compliance with the Helsinki Declaration and all participants gave written, informed consent.

All children born in 1921 and 1936 and attending school in Scotland on 1^st^ June 1932 or 4^th^ June 1947 respectively were tested at age 11 (±0.5) years for general cognitive ability [26]. The test administered was a version of the Moray House Test No. 12, which was concurrently validated against the Terman-Merrill revision of the Binet Scales with a coefficient of approximately 0.8. At age 64(±1) years (ABC36) and age 78(±1) years (ABC21), crystallised cognitive ability was assessed by the National Adult Reading Test (NART) [27] which tests word knowledge; and fluid, non-verbal reasoning ability was assessed by Raven’s Progressive Matrices [28]. Participants also gave blood samples at this age, and weight and height were measured by trained research nurses. The Scottish Index of Multiple Deprivation (SIMD) decile was used as the measure of socioeconomic circumstances at the time of blood sampling.

Tissues were sampled from 4 brains donated to the Aberdeen Brain Bank post-mortem. These were not from ABC participants, however the donors were from the same region and were approximately contemporary with the ABC participants. Brain 1 was from a male with typical Alzheimer’s disease aged 65 years, brain 2 from a mixed Alzheimer’s and cerebral small vessel disease (CSVD) male aged 75 years, and brains 3 and 4 were from two control samples, both male, aged 67 and 70, with no evidence of neurodegeneration beyond the normal expectation for that age. Samples were taken in duplicate from the anterior hippocampus (AH), basal ganglia (BG), posterior hippocampus (PH), periventricular white matter (PWM) and thalamus (TH) of each brain with sterile scalpel and forceps. Genomic DNA was extracted using EZ1 DNA Tissue kits (Qiagen, Crawley, UK) and bisulphite conversion using Zymo EZ DNA Methylation-Gold Bisulfite kits (Zymo Research, California, USA).

Genomic DNA was extracted from lymphocytes using QIAamp DNA Mini Blood kits (Qiagen, Crawley, UK) and DNA methylation was measured by pyrosequencing using a PyroMark MD system (Qiagen, Crawley, UK) following bisulphite conversion of DNA using Zymo EZ DNA Methylation-Gold Bisulfite kits (Zymo Research, California, USA). Bisulphite conversion efficiency was evaluated by including a non-CpG cytosine base as a conversion control in each assay.

DNA methylation was measured in nine imprints–*IGF2*, *H19ICR*, *SNRPN*, *PEG3*, *MEST1*, *NESPAS*, *KvDMR*, *IG-DMR* and *ZAC1*. Assays were carried out using previously published primer sets when these were available; *IGF2* [29], *PEG3* [30], *SNRPN* [31], *KvDMR*, *IG-DMR* and *ZAC1* [10]. Avoidance of SNPs is one of the criteria for assay designs. In the previously developed *ZAC1* assay, methylation site 8 of 11 corresponded to a SNP (rs77574073) and this site was excluded from the analyses. FASTA sequences were used to design the *H19ICR* assay in house using PyroMark Assay Design Software (version 2.0, Qiagen, Crawley, UK). *MEST1* and *NESPAS* primer sets were designed by Dr Mitsutero Ito at Cambridge University. The following primers were used; *H19ICR* assay: forward 5’-TGGGGATTTTGATGGGGTTA-3’, reverse 5’-biotin-CCTACTCCAAACATTATAAAAAAAACTAAC-3’ and sequencing primer 5’-GATGGTTAGGGTGTGTT-3’; *MEST1* assay: forward 5’-AAGGGGGTTTTGTTTTTTTAATTGTG-3’ [10], reverse 5’-biotin-AAACTCTATTAAACCCACCACCAAACTAAT-3’ and sequencing primer 5’-TTGTTGTAAAGGAAATTT-3’; *NESPAS* assay: forward 5’-TGTGTATATATTAAGGTTATTAGGTG-3’, reverse 5’-biotin-TAATCAATCAACTCCTTTAACCCC-3’ and sequencing primer 5’-GGTTAGTTTTGAGTTTAT-3’. The genomic locations of the assays are shown in S1 Table.

All the assays met our criteria for conversion efficiency and there was no correlation between this and any of the imprint methylation results. The technical replication in our assays is such that routine repeat analyses have a minimal effect on study power therefore methylation in samples is determined singly. Outliers and individual data points with significant leverage on the results are assessed and, where appropriate, reanalysed but this is rarely required. All of the assays included adjustment for plate run to avoid any batch effects during the methylation analysis. Plate was included as a factor in all the regression analyses. The methylation level at adjacent CpG sites within each imprint was significantly correlated (p<0.001) in all the genes studied and an average methylation level for each imprint was used in the analysis. The number of CpG sites averaged was 4 for *SNRPN* and *IGF2*, 5 for *IG-DMR*, 6 for *H19ICR* and *MEST1*, 7 for *PEG3*, 8 for *NESPAS*, 10 for *ZAC1* and 12 for *KvDMR*.

Statistical analysis was carried out using STATA/MP version 15 (Stata Corp, College Station, Texas, USA). Multivariate linear regression was carried out independently for each imprint with adjustment for sex and adult socioeconomic circumstance. Methylation was the explanatory variable in all regressions. Outliers with potentially high leverage on regression outcomes were re-analysed. Where the 5% statistical significance cut-off was observed in the cohort as a whole, the regressions were also stratified by sex and tested for an interaction term between methylation and sex by ANOVA in the combined cohorts. Cohort was included as an additional factor in the combined cohort regressions. The proportion of variance accounted for in models (adjusted R^2^) is reported for significant results.

## Results

Imprint methylation data obtained for ABC36 participants are described along with the participant cognitive test scores in Table 1. There were no significant sex differences in MHT, NART or Raven’s test scores. The ABC36 cohort consisted of 50.5% females. Mean weights were 67.5 (SD 12.7) kg in females and 79.3 (SD 11.4) kg in males, (p<0.001). Mean heights were 158.9 (SD 6.1) cm in females and 171.9 (SD 6.0) cm in males, (p<0.001). Mean BMI was 26.8 (SD 4.9) kgm^-2^ in females and 26.8 (SD 3.5) kgm^-2^ in males and the difference was not statistically significant.

**Table 1:** ABC36 cognitive test scores and imprint methylation levels in blood DNA.

	Mean	Standard Deviation	n
^1^**Measures of cognitive ability**			
**Moray House Test**	42.0	13.5	480
**National Adult Reading Test**	108.9	10.6	474
**Raven’s Progressive Matrices**	35.6	8.8	462
^2^**Average methylation**			
***H19ICR***	58.0	4.3	462
***IGF2***	49.3	5.8	467
***SNRPN***	46.6	3.7	464
***PEG3***	51.2	2.8	474
***MEST1***	48.2	3.1	469
***NESPAS***	47.8	2.9	472
***KvDMR***	47.8	2.7	461
***IG-DMR***	63.1	1.8	478
***ZAC1***	43.8	3.6	477

^1^Cognitive ability was measured in children using the Moray House Test (a test of general intelligence), and in adults using the National Adult Reading Test (a measure of crystallised intelligence) and Raven’s Matrices (a measure of fluid intelligence).

^2^Mean methylation across measured CpG sites within each imprint.

Imprint methylation data obtained for ABC21 participants are described along with the participant cognitive test scores in Table 2. There were no significant sex differences in MHT, NART or Raven’s test scores. The ABC21 cohort consisted of 47.5% females. Mean weights were 64.0 (SD 13.0) kg in females and 72.4 (SD 10.0) kg in males, (p<0.001). Mean heights were 156.3 (SD 5.8) cm in females and 167.5 (SD 6.6) cm in males, (p<0.001). Mean BMI was 26.4 (SD 4.8) kgm^-2^ in females and 25.8 (SD 3.2) kgm^-2^ in males and the difference was not statistically significant.

**Table 2:** ABC21 cognitive test scores and imprint methylation levels in blood DNA.

	Mean	Standard Deviation	n
^1^**Measures of cognitive ability**			
**Moray House Test**	37.9	13.1	204
**National Adult Reading Test**	110.1	9.4	167
**Raven’s Progressive Matrices**	26.5	8.6	167
^2^**Average methylation**			
***SNRPN***	42.9	3.3	220
***MEST1***	43.2	3.9	201

^1^Cognitive ability was measured in children using the Moray House Test (a test of general intelligence), and in adults using the National Adult Reading Test (a measure of crystallised intelligence) and Raven’s Matrices (a measure of fluid intelligence).

^2^Mean methylation across measured CpG sites within each imprint.

No significant associations were identified between childhood or adult cognitive test scores and weight, BMI or analysis plate with test scores as the dependent variable. Raven’s test score was associated with height in ABC21 (+0.177 95% CI 0.019,0.336; p = 0.029; R^2^ = 0.026) and in ABC36 (+0.127 95% CI 0.039,0.215; p = 0.005; R^2^ = 0.015). All cognitive test scores were positively associated with SIMD in ABC21 (MHT: +0.734 95% CI 0.042,1.426; p = 0.038; R^2^ = 0.018; NART: +1.230 95% CI 0.711,1.749; p<0.001; R^2^ = 0.122; Raven’s: +0.538 95% CI 0.042,1.034; p = 0.034; R^2^ = 0.023) and ABC36 (MHT: +1.385 95% CI 0.995,1.776; p<0.001; R^2^ = 0.093; NART: +1.227 95% CI 0.923,1.530; p<0.001; R^2^ = 0.120; Raven’s +0.854 95% CI 0.597,1.112; p<0.001; R^2^ = 0.085). All of the associations between methylation and cognition were adjusted for SIMD, and adjustment for height did not change the significance of Raven’s associations with methylation.

The methylation levels within the imprinted genes were all close to the 50% level characteristic of imprints and within the range of 33–70% typically reported for imprints [32] (Tables 1 and 2). There was no evidence of a difference in the level of methylation for the imprints between sexes with the exception of *H19ICR* methylation in ABC36 which was significantly higher in females (-1.086 95% CI -1.881,-0.290; p = 0.008; R^2^ = 0.003). There was no evidence of an association between the level of methylation and SIMD with the exception of *H19ICR* methylation in ABC36 (-0.175 95% CI -0.310,-0.040; p = 0.011; R^2^ = 0.012). *KvDMR* (+0.093 95% CI 0.036,0.149; p = 0.001; R^2^ = 0.020) and *MEST1* (-0.078 95% CI -0.144,-0.012; p = 0.020; R^2^ = 0.009) methylation were associated with BMI in ABC36. Adjustment of the *MEST1* associations with cognition for BMI did not change the level of significance. No further associations were observed between methylation and weight, BMI, height or SIMD.

**Fig 1:**
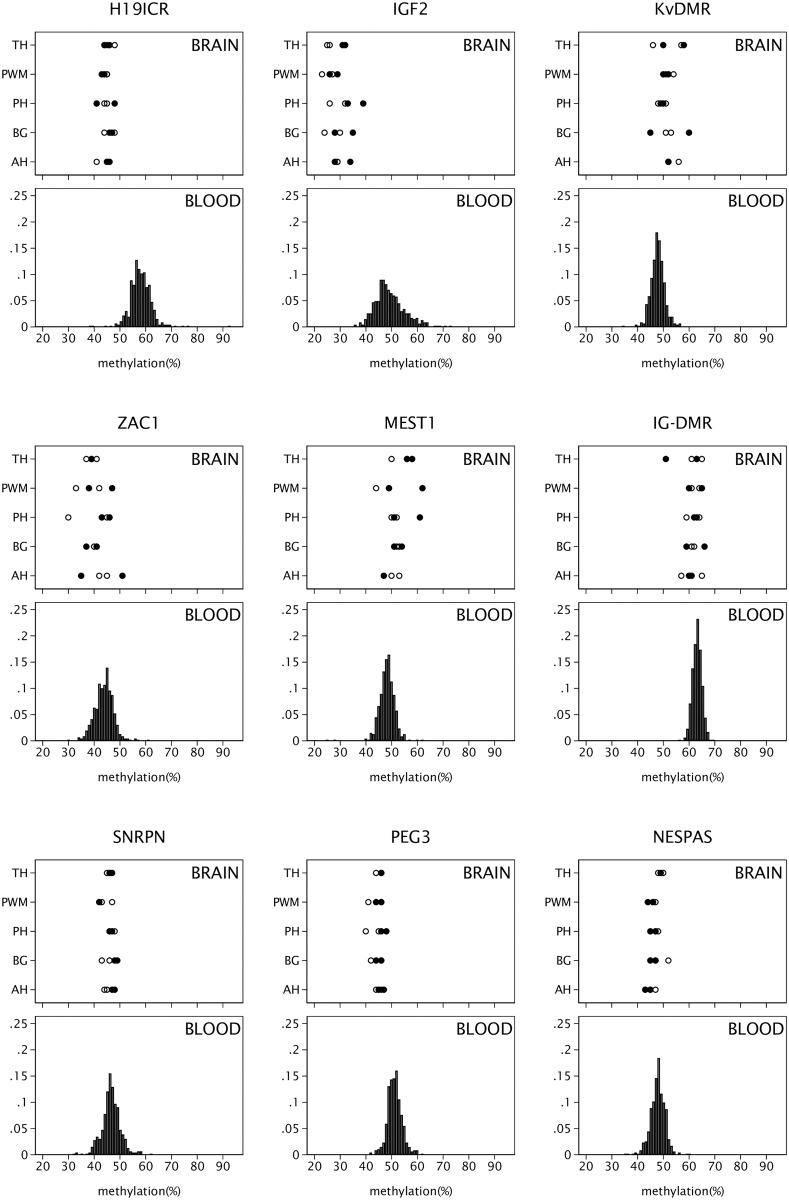
Brain and blood methylation comparison. DNA methylation in the imprints *H19ICR*, *IGF2*, *IG-DMR*, *KvDMR*, *MEST1*, *NESPAS*, *PEG3*, *SNRPN* and *ZAC1* in blood from ABC36 (n = 485) and five selected regions from four brains sampled post-mortem (AH: Anterior Hippocampus. BG: Basal ganglia. PH: Posterior Hippocampus. PWM: Periventricular white matter. TH: Thalamus). The individual methylation values for each brain sample are shown with cases (individuals with evidence of Alzheimer’s Disease) represented by open circles and controls (no apparent neurodegeneration beyond that expected for age) by closed circles. The population distribution of methylation levels in blood are shown on the same methylation scale.

Methylation status for the nine imprints in the cohort blood samples were compared with values in a range of anatomical sites (anterior hippocampus, posterior hippocampus, periventricular white matter, and thalamus) in post-mortem samples from the brains of four adults from the same geographical location (Fig 1). The number of brains studied was small but the results demonstrate that the methylation levels for each imprint were similar for all of the brain anatomical sites studied and there was no separation by Alzheimer’s Disease status. This is consistent with the fact that none of the imprinted loci studied here have been reported to differ by Alzheimer’s Disease status [33]. In addition, the overall level was similar to that in the blood, with the exception of *H19ICR* and *IGF2*. In the majority of the imprints the methylation in the brain was similar to the 50% level expected for imprinted genes but for *IGF2* in particular the brain methylation level was lower at around 30%. Some methylation signatures differ between blood cell types but the imprinted loci studied here were not identified as differentially methylated by cell type in studies using the 450k array [34]. No comparison was possible for the *IG-DMR* locus as this was not present on the 450K array but it was notable there was agreement between blood and brain for this imprint, though the level of methylation was high at around 60%, and none of the results reported here for *IG-DMR* and the outcomes of interest were significant.

The relationships between imprinting methylation and measures of cognitive ability in childhood and adulthood are shown in Table 3. The results are expressed as score point change per methylation %. In the ABC36 cohort, *SNRPN* methylation was positively related to childhood MHT score (+0.335 95%CI 0.008,0.663; p = 0.045; R^2^ = 0.097) and NART score in adults (+0.262 95% CI 0.007,0.517; p = 0.044; R^2^ = 0.127). The *MEST1* methylation was negatively related to childhood MHT score only (-0.416 95% CI -0.792,-0.041; p = 0.030; R^2^ = 0.106). The association between *SNRPN* and NART was no longer significant when adjusted for childhood MHT, though this might be expected as the childhood MHT score was highly correlated with the adult scores for NART (+0.575 95% CI 0.526,0.623; p = 0.000; R^2^ = 0.534) and Ravens (+0.403 95% CI 0.355,0.452; p = 0.000; R^2^ = 0.367). If a False Discovery Rate of 25% is applied to the full 9 MHT imprints tested the significant p values are retained.

**Table 3:** DNA methylation in imprints and measures of childhood and adult cognitive ability in ABC36.

	Moray House Test		National Adult Reading Test		Raven’s Progressive Matrices	
Imprint	Coefficient [95% CI]	p value	Coefficient [95% CI]	p value	Coefficient [95% CI]	p value
***H19ICR***	-0.104 [-0.383,0.176]	0.466	-0.103 [-0.321,0.116]	0.355	0.045 [-0.141,0.230]	0.635
***IGF2***	0.026 [-0.182,0.233]	0.809	0.151 [-0.009,0.312]	0.065	0.042 [-0.095,0.179]	0.544
***SNRPN***	0.335* [0.008,0.663]	0.045	0.262* [0.007,0.517]	0.044	0.195 [-0.025,0.416]	0.083
***PEG3***	-0.008 [-0.430,0.415]	0.971	-0.040 [-0.369,0.289]	0.810	-0.182 [-0.465,0.102]	0.209
***MEST1***	-0.416* [-0.792,-0.041]	0.030	-0.184 [-0.480,0.111]	0.220	-0.071 [-0.329,0.186]	0.587
***NESPAS***	0.008 [-0.396,0.411]	0.970	0.065 [-0.251,0.381]	0.687	0.112 [-0.161,0.385]	0.419
***KvDMR***	-0.123 [-0.568,0.323]	0.589	-0.217 [-0.563,0.128]	0.217	0.033 [-0.262,0.327]	0.827
***ZAC1***	-0.117 [-0.449,0.215]	0.489	-0.116 [-0.371,0.139]	0.373	0.031 [-0.185,0.247]	0.779
***IG-DMR***	-0.036 [-0.684,0.613]	0.914	0.110 [-0.398,0.617]	0.671	0.207 [-0.222,0.636]	0.344

Multivariate linear regression was carried out with imprint methylation as the explanatory variable and cognitive ability at age 11 and in adulthood as the dependent variable for each imprint in ABC36. Cognitive ability was measured at age 11 using the Moray House Test (a test of general intelligence), and in adults using the National Adult Reading Test (a measure of crystallised intelligence) and Raven’s Progressive Matrices (a measure of fluid intelligence). All regressions were adjusted for sex, adult socioeconomic status (index of multiple deprivation decile) and plate. The results are expressed as score point change per methylation %. P values are not adjusted for multiple testing correction.

* p<0.05.

**Fig 2:**
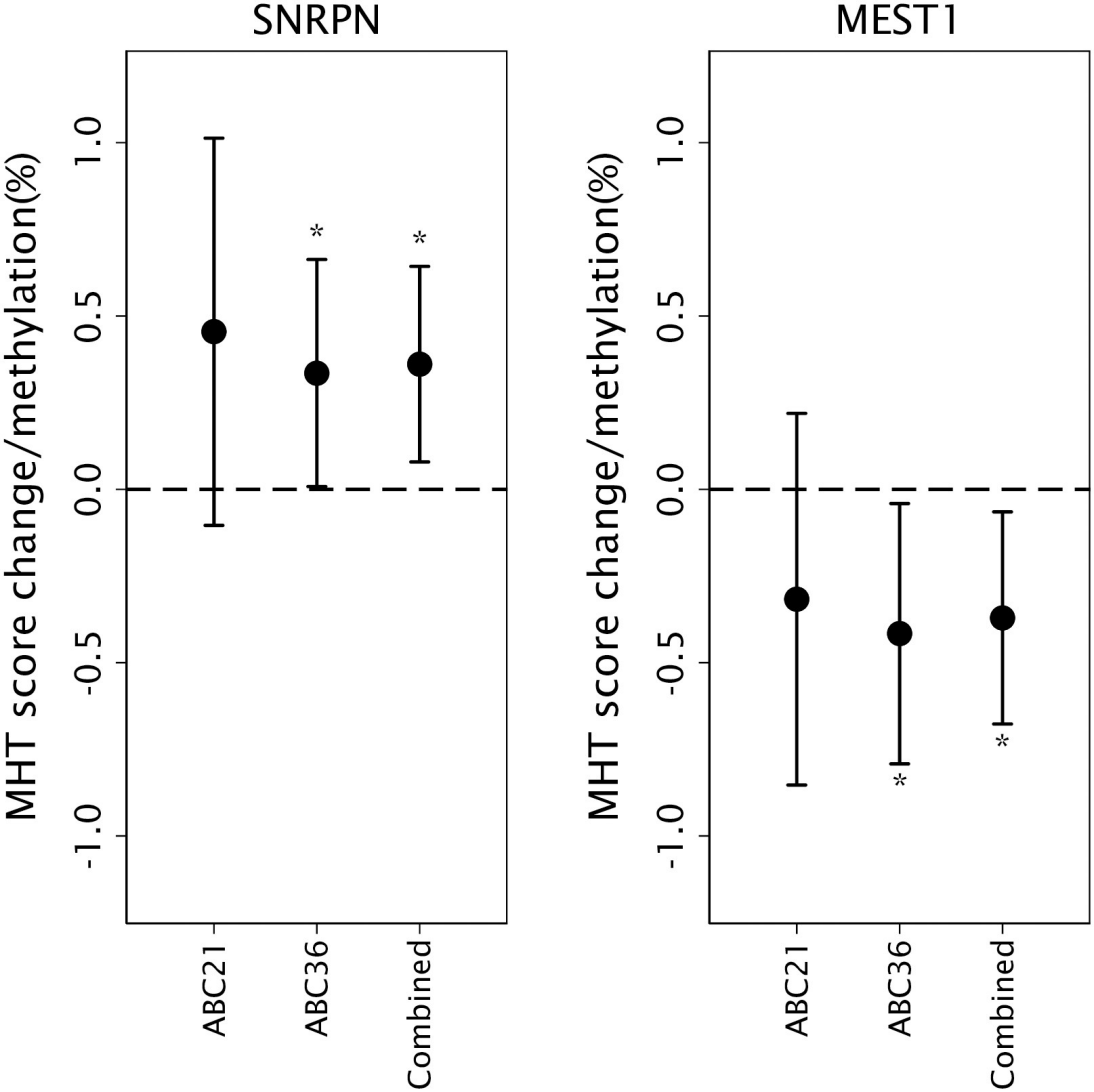
*SNRPN* and *MEST1* methylation in ABC cohorts. Regression coefficients with 95% CIs from regressions between *SNRPN* and *MEST1* methylation in ABC36, ABC21 and the combined cohort and childhood cognitive ability measured at age 11 using the Moray House Test (MHT). All regressions were adjusted for sex, adult socioeconomic circumstance (index of multiple deprivation decile), plate and the combined cohort was additionally adjusted for cohort.

Further analysis of the significant ABC36 findings in the ABC21 cohort alone indicated that the effect size and direction were similar for *SNRPN* (MHT +0.455 95% CI -0.104,1.013; NART +0.182 95% CI -0.231,0.594) and *MEST1 (*MHT -0.317 95% CI -0.853,0.219) (Fig 2). The ABC21 cohort was around half the size of ABC36 and the results in ABC21 alone were not significant but in the combined cohorts the MHT results were more significant for both *SNRPN* (+0.361 95%CI 0.079,0.643; p = 0.012; R^2^ = 0.090) and *MEST1* (-0.371 95%CI -0.677,-0.065; p = 0.017 R^2^ = 0.096); regressions additionally adjusted for cohort (Fig 2).The *SNRPN* association with NART was also more significant in the combined analysis (+0.241 95% CI 0.025,0.457; p = 0.029; R^2^ = 0.137) but, as with ABC36 alone, the association was not significant with adjustment for childhood MHT.

There was no evidence of an interaction with sex for the *SNRPN* effect on MHT in ABC36 alone or the combined cohort. There was a significant interaction with sex for *MEST1* and MHT in ABC36 alone (p = 0.023), though the effect was attenuated in the combined cohort analysis (p = 0.063). Further analysis of the sex interaction in *MEST1* indicated a highly significant result in males for MHT in ABC36 alone (-0.929 95% CI -1.531,-0.326; p = 0.003; R^2^ = 0.152) and in the combined cohort analysis (-0.807 95% CI -1.268,-0.346; p = 0.001; R^2^ = 0.138).

## Discussion

Methylation of the paternally expressed, maternally methylated genes *SNRPN* and *MEST1* in adulthood was associated with cognitive ability in childhood in a longitudinal cohort. The childhood cognitive associations with *SNRPN* were positive in both sexes, while the associations with *MEST1* were negative and strongest in males. This is the first report of an association between *SNRPN* and *MEST1* imprinting methylation and cognitive ability in a normal population. Some strengths and limitations of the current study should be noted. This longitudinal cohort study provides invaluable cognitive data from single individuals across the life course. The numbers in the analyses are effectively halved when considering the sexes separately but this is more likely to result in false negatives, due to reduced power, than in false positives. We did not adjust for multiple testing, but we checked the consistency of significant results in a second older cohort and carried out a combined cohort analysis with adjustment for cohort membership. Participants from both cohorts were recruited from a small geographical area and were born in the same year, 1921 or 1936. They undertook the same childhood cognitive test at the same age, and had the same information collected following recruitment. In terms of wider significance, imprinting is an established biological process that is essential in normal development and therefore the factors that influence imprinting are likely to operate via conserved mechanisms across populations.

Although the post-mortem brain sample size is small, the inclusion of the same imprint methylation analysis, using the same assays, in brain samples is a strength of the study as it demonstrates similar methylation profiles in different cell types and brain regions and the assays in which it is valid to assume similar levels in blood and brain. In all but two of the assays (*H19ICR* and *IGF2*) methylation levels in blood and in five separate anatomical regions of brain were similar to each other and similar to methylation levels in the same genes in cord blood at birth in a contemporary cohort [6,8]. Tissue-specific methylation patterns are sometimes observed in secondary, or somatic DMRs, of which the *IGF2* imprint is an example, indicating that differential methylation was established post-fertilization and therefore may not be inherited by all tissues [4]. Establishment of secondary DMRs requires the presence of a ubiquitous germline DMR [35] and in the case of *IGF2*, this is the *H19* imprint control region (ICR).

Evidence from imprinting syndromes supports a role for *SNRPN* in cognitive ability. Prader-Willi syndrome (PWS; OMIM 176270) and Angelman syndrome (AS; OMIM 105830) are clinically distinct imprinting disorders characterised primarily by neurological and behavioural features [21]. These conditions can result from microdeletion, uniparental disomy, and epigenetic changes in the 15q11-q13 chromosomal region [21,22,36,37]. Imprinting of 15q11-q13 is regulated by an imprinting control centre which contains a differentially methylated region at the 5’ end of *SNRPN* that controls expression throughout 15q11-q13 [38] and which was assayed here. *SNRPN* has been proposed as the most likely candidate gene [21,22] for PWS, which results in an approximately normal distribution of IQ but with a mean 40 points below that of the general population, with the IQ of unaffected siblings being correlated with that of the PWS cases [22,37,39]. Autism spectrum disorders with varying degrees of learning disability have also been linked to duplication of chromosome region 15q11q-13 [40]. Epigenetic inheritance from fathers may also be relevant as the risk of autism in human offspring is predicted by epigenetic differences in genes within the *SNRPN* containing gene cluster in sperm [41].

A pathway for *SNRPN* involvement in postnatal brain development has recently been identified, where *SNRPN* expression directly regulates the expression level of the nuclear receptor *Nr4a1* which is critical for the proper development of cortical neurons [42]. Overexpression of *SNRPN* results in increased dendritic spine density, and morphological characteristics of dendritic spines contribute to cognitive ability [43]. This presents a potential pathway by which imprint methylation may impact brain function but more research is needed on the mechanism.

A third imprinting syndrome, Silver–Russell Syndrome (SRS; OMIM 180860) has been associated with changes in several chromosomal regions, including 11p15 [44]—a region which contains an imprinting cluster regulated by two ICRs, *H19ICR* and *KvDMR1*—and the 7q31 [45,46] which contains *MEST1*. Pathological cognitive impairment is not generally associated with SRS but a sibling controlled study investigating cognitive development in SRS associated with chromosome 7 imprinting regions, including *MEST1*, found a significant difference in IQ in direct analysis of paired differences between the subsample of children with SRS and a non-affected sibling [47]. In animal studies, *MEST1*-deficient female mice demonstrated impaired maternal care for their offspring, suggesting a link between *MEST1* imprinting and brain function [19]. A role for *MEST1* imprinting in human cognition is further supported by genome-wide association, which suggests an autism susceptibility locus within the *MEST1* containing chromosomal region 7q31-q32 in males on the autism spectrum [24]. *MEST1* methylation has also been associated with maternal stress exposure *in utero [48]*, raising the possibility that the association between *MEST1* methylation and cognitive ability may reflect a more general response to *in utero* exposure to stress.

The stronger effect of *MEST1* in males was notable. Maternal stress has been reported to influence *MEST1* methylation in opposite directions in male and female offspring [48]. Furthermore, the effect of the genomic changes in the *SNRPN* containing PWS region on intelligence was different in magnitude in males and females. The correlation between the intelligence of PWS offspring and that of their mothers in males was found to be over four times that observed in female offspring, and two fold higher in a selected sub-group of typically developing children [37]. Furthermore, a sex-specific association between maternal depression and *PEG3* expression has been reported in the placenta of male, but not female, offspring [49]. The mechanisms underlying these sex effects are not understood. The difference in the direction of significant association for *SNRPN* and *MEST1* is also noteworthy. This observed difference in direction, and an opposite direction of response in imprinted gene methylation to folic acid [6] suggest that the regulation of methylation is not necessarily uniform across all imprints.

The associations were strongest between *SNRPN* and *MEST1* and a childhood measure of cognition (MHT). Of the tests taken in later life, the imprint-cognition associations were stronger for measures of crystallised (NART) rather than fluid (Raven’s) intelligence. These results were no longer significant when adjusted for MHT but the MHT score is itself highly correlated with NART and Raven’s. Crystallized abilities (such as word knowledge) depend more on experience and knowledge and increase throughout young adulthood and middle-age, then plateau, showing little or no further gain into old age [50]. Fluid abilities show a pattern of increase throughout young adulthood, followed by slow decline beginning in middle-adulthood which continues throughout old age [50].

The methylation changes observed were subtle but comparable in magnitude to those observed in similar studies, for example, in response to prenatal obesity [51,52], prenatal exposure to famine [53], and periconceptual folic acid intake [6]. It is worth noting that large change in methylation at imprint loci typically results in pathology and that was not the focus of this study. Whilst these changes are modest they may have significant effects. Previous analysis in cord blood has suggested that a 1% change in methylation at the *IGF2* DMR in response to prenatal exposure to cigarette smoke, corresponded to an approximately 2-fold change in *IGF2* transcription [54]. There is also the potential for small changes in imprinting methylation to influence the wider genome through long range interactions over thousands of bases.

The magnitude of the cognitive effects can be illustrated by translating the key childhood cognitive ability score into the commonly used IQ type scale, although it should be noted that we are not reporting IQ values. A 1% change in *SNRPN* and *MEST1* methylation translates into a 0.4 point change in IQ type scale whilst two standard deviations (approximately full range) in the population distribution of *SNRPN* and *MEST1* translates into around 3 points on the IQ type scale. The *SNRPN* methylation assay used here was developed as a diagnostic test for PWS and AS [31], which manifest predominantly as cognitive phenotypes, and the methylation level of *SNRPN* in blood from the cohort studied here was similar to that in five different brain anatomical locations (including two hippocampal regions) measured in post-mortem samples. The levels of *SNRPN* methylation reported here in later life are also similar to those measured in cord blood in a large contemporary birth cohort from the same city [6]. Cross sectional studies have demonstrated stability in children over the first 7 years of life [7] and in adult females between age 25 years and 85 years [55]. This evidence supports the conclusion that, for the regions reported here, the imprints are stable once set.

Imprints can be influenced by both genetics and the environment. For genetic effects, epigenetic states may be influenced by variation in the sequence underlying the epigenetic regions being measured [56], in proximal elements [57], or in the epigenetic machinery involved in setting and maintaining the methylation pattern. The *SNRPN* and *MEST1* assays used here specifically excluded genetic variants within the region measured and we have no evidence for a genetic effect underlying the epigenetic associations with cognitive ability but this remains a possibility that cannot yet be ruled out. There is evidence that both *SNRPN* and *MEST* methylation may be influenced by the early environment. Animal studies indicate that paternal alcohol intake influences *SNRPN* and *PEG3* methylation in the brains of their offspring [20], while human studies have shown that S*NRPN* methylation is altered in children conceived by intracytoplasmic sperm injection to overcome male infertility [7] and is influenced by the IVF culture media used [58]. Maternal stress during pregnancy has been associated with higher infant DNA methylation at the *MEST* DMR with some evidence for a sex bias in the response [48] Folate intake during pregnancy is linked to *IGF2* and *PEG3* but not *SNRPN* methylation [6] indicating that sensitivity to the early environment depends on the timing and nature of the environmental exposure.

The findings reported here are consistent with the known importance of the *SNRPN* containing 15q11-q13 and the *MEST1* containing 7q31-34 regions in cognitive function. These findings, and their sex specific nature in *MEST1*, point to new mechanisms through which complex phenotypes such as cognitive ability may be inherited and the potential role of the environment in modifying that heritability. The process of epigenetic imprinting—within *SNRPN* and *MEST1* in particular—and the factors that influence it, are worthy of further study in relation to the determinants of cognitive ability.

## Supporting information

S1 Table Genomic locations of pyrosequencing assays in *Homo sapiens* genome v37. Chromosome, start and end coordinates for each differentially methylated region (DMR) are tabulated.
(DOCX)

S1 Data ABC21 and ABC36 methylation data. Methylation levels at *H19ICR*, *IGF2*, *SNRPN*, *PEG3*, *MEST1*, *NESPAS*, *KvDMR*, *ZAC1* and *IGDMR1* imprints determined by pyrosequencing in blood samples from the ABC21 and ABC36 cohorts alongside cohort (ABCgroup), sex and analysis plate.
(XLSX)
