# Correction: Imprinting methylation in *SNRPN* and *MEST1* in adult blood predicts cognitive ability

**DOI:** 10.1371/journal.pone.0215422

**Published:** 2019-04-10

**Authors:** Marlene Lorgen-Ritchie, Alison D. Murray, Anne C. Ferguson-Smith, Marcus Richards, Graham W. Horgan, Louise H. Phillips, Gwen Hoad, Ishbel Gall, Kristina Harrison, Geraldine McNeill, Mitsuteru Ito, Paul Haggarty

## Notice of republication

An incorrect version of S1 Data was published in error. This article was republished on April 3, 2019 to correct for this error. In addition, the article’s Data Availability statement has been updated to reflect this change. Please download this article again to view the correct version.
