# Supplementary material for: Imprinting methylation in SNRPN and MEST1 in adult blood predicts cognitive ability
Source: PLoS One. 2019 Feb 1;14(2):e0211799. doi: 10.1371/journal.pone.0211799 (PMC6358095; doi:10.1371/journal.pone.0211799)
Supplement: S1 Table — Chromosome, start and end coordinates for each differentially methylated region (DMR) are tabulated. (DOCX) [file pone.0211799.s001.docx]

| DMR | Chromosome | Start | End |
| --- | --- | --- | --- |
| H19ICR | 11 | 2020437 | 2020625 |
| IGF2 | 11 | 2154263 | 2154457 |
| IGDMR | 14 | 101275674 | 101275982 |
| KvDMR | 11 | 2721925 | 2722179 |
| MEST1 | 7 | 130131093 | 130131345 |
| NESPAS | 20 | 57426667 | 57426924 |
| PEG3 | 19 | 57351944 | 57352095 |
| SNRPN | 15 | 25200013 | 25200250 |
| ZAC1 | 6 | 144329449 | 144329712 |
